# Medical Education

**Published:** 1872-01

**Authors:** 


### MEDICAL EDUCATION.

We have been informed and requested to state, that the Trustees and Faculty of the Atlanta Medical College, at the instance of the latter, have determined to change the course of instruction of the institution, and to meet, hereafter, the most extreme demands of the profession for a higher medical education. The details of the plan have not as yet been definitely settled; but so much has been decided as to make it *certain* that the regular graduating period will be at the end of the winter course—two winter courses of lectures being required. The writer of this article is in favor of the *graded* system of medical education, running through a period of at least two years of continuous (except short vacations) college and hospital instruction to those who have had one year of private pupilage, and a three years' course of a similar character to those who commence their medical education in the college. To our mind, there can scarcely be a greater absurdity than the proceedings of even our best colleges, up to the present time, notwithstanding all the volumes that have been spoken and written upon this subject for the last twenty years. We look with great interest to the plan recently adopted by the medical department of Harvard University, and hope that the success may be such as not only to encourage its prosecution as the permanent policy of that institution, but that it may be adopted by every medical college in the United States.

